# Sleep Pattern and Problems in Young Children Visiting Outpatient Department of a Tertiary Level Hospital in Kathmandu, Nepal

**DOI:** 10.1155/2020/8846288

**Published:** 2020-12-02

**Authors:** Shrijana Pandey, Shristi Bhattarai, Anwesh Bhatta

**Affiliations:** ^1^Department of Nursing, Kathmandu Medical College, Nepal; ^2^Department of B.Sc. Nursing, Kathmandu Medical College, Nepal; ^3^Department of Pediatrics, Kathmandu Medical College, Nepal

## Abstract

**Background:**

Sleep is an important parameter of a child's growth and development. The pattern and duration of sleep varies with age. Sleep problems are a common occurrence during childhood days, and these problems that establish in childhood are presumed to continue later in life. Many times, parental concerns regarding their child's sleep problems like difficulty in putting to sleep, frequent night time awakening, and waking up early are overlooked during their visits to the hospital.

**Objective:**

The aim of this study was to find out the sleep patterns and problems of children aged six to thirty-six months. *Methodology*. A cross-sectional study was conducted at the pediatric outpatient department of Kathmandu Medical College Teaching Hospital from October, 2019 till March, 2020. Two hundred and forty-nine respondents were chosen purposively and were given questionnaires to be filled out. Research instrument was a standard, Nepali version of a structured questionnaire called Brief Infant Sleep Questionnaire (BISQ) which contained questions related to sleep parameters and sleep problems existing among young children of 6-36 months. Mean, standard deviation, frequencies, and Kruskal Wallis test were used for statistical analysis.

**Results:**

The mean duration of total sleep was 12.12 ± 2.00 hours, while that of night sleep was 9.22 ± 1.19 hours and mean daytime nap was 2.90 ± 1.66 hours. Most of the children (96%) coslept with their parents, and 55% of the children had feeding as a bedtime ritual. Overall, 19.6% of the children had sleep problems as identified by BISQ although only 5.6% of the parents perceived that their children had it.

**Conclusions:**

Sleep problems were present among young Nepalese children included in our study, and sleep assessment should be a part of every health checkup for children.

## 1. Introduction

Sleep is an integral part of a child's health and development. During the period of sleep, development of higher cognitive function occurs rapidly in the brain, and among parents, concerns regarding child's sleep are ranked as the fifth leading concern [1]. Sleep pattern varies with age, and it is strongly influenced by environmental and cultural factors [2]. Moreover, a study carried out in six different countries of Southeast Asia has shown that there are variations in the sleep characteristics of young children [3]. The duration of total sleep and the frequency and duration of nighttime awakening have been found to decrease as the child grows up, whereas the longest sleep duration and duration of sleep at night has been found to increase with age [4].

Sleep disorders are very common in childhood, and they pose significant consequences for both children and parents. Behavioral sleep disorders may manifest as sleep onset delay, sleep interruptions, early morning awakening, or a combination of these [5]. Research studies have shown that sleep problems are common in up to 10.5% in toddlers, and the most common problem in these young children is difficulty in falling asleep [6]. Sleep problems, consequently, can lead to inattention [7, 8] and hyperactivity among children leading to daytime sleepiness [7].

Sleep problems like bed time resistance and frequent night awakenings can affect the healthy sleep cycle in children [9]. A study suggests that 21% of the sleep problems that arise during infancy can persist up to 36 months [10]. A research study carried out in Lalitpur, Nepal, showed that 20.3% of young children have sleep problems [11]. Furthermore, the duration of night time sleep seems to be lower in developing countries like Nepal and India as compared to developed countries [11, 12].

Sleep problems, however, are many times underreported and neglected by physicians and parents in common practice [9]. As research in this important aspect of young child's health has been very few in Nepal, the researchers were interested to carry out this study using a standard, validated Nepali version of Brief Infant Sleep Questionnaire (BISQ). BISQ has been proven to be an effective tool for use in clinical and research settings [13]. The focus of the present study was to assess the pattern of sleep and sleep problems in young children visiting the pediatric outpatient department of Kathmandu Medical College.

## 2. Methodology

A descriptive cross-sectional study was carried out among primary caregivers of children aged 6-36 months from October, 2019 to March, 2020. Ethical clearance was obtained for the study from the institutional review committee of Kathmandu Medical College (Ref: 181020193). The ethical principles as stated in the Declaration of Helsinki were taken into account while conducting this study. A written informed consent was obtained from all the respondents to ensure their willingness to participate. The participation in the study was voluntary and respondents were identified using code numbers. Data was collected using a purposive sampling technique at the pediatric outpatient clinic of Kathmandu Medical College. The inclusion criteria was that the child had to be of age between 6 and 36 months should be visiting a hospital for general health checkups (immunization, growth/development assessment, or general health-related queries particular to the child's age) and should not be currently under stimulant or medications that potentially affect sleep. Similarly, the respondents included had to be primary caregivers, who had a major responsibility of looking after the child and lived together with the child. Taking the prevalence of sleep problems among young children as 20.3% [11], 249 respondents were recruited from the study site using statistical formula of *n* = *z*2*pq*/*d*^2^, where *d* (allowable error) = 0.05, *z* (confidence level) = 1.96, *p* (prevalence of sleep problem) = 0.203, and *q*(1 − *p*) = 0.797.

The research instrument included questions related to sociodemographic variables and sleep pattern and problems of the child. The sleep characteristics were assessed by using Nepali version of the BISQ. BISQ is a standard tool used for the assessment of sleep pattern, characteristics, and parental perception of sleep problems [14]. This tool has been translated into various languages like Brazilian, Spanish, Turkish, Portugese, and Nepali. Specifically, BISQ assess sleep parameters like place and position of sleep, bedtime rituals, duration of day and night sleep, and frequency and duration of night time awakening. The Nepali version of BISQ has been reported to be useful for researches in assessing sleep problems in infants and toddlers [15]. For collecting data, respondents were given the questionnaire to be filled out by themselves. When the respondents were found to be illiterate, the researcher read out the questionnaire for them and filled out the responses. Collected data was checked for its completeness and accuracy. Data was then edited, coded, and entered into SPSS software-20. Descriptive statistics like mean, frequencies, percentages, standard deviation, and Kruskal Wallis *H* were used for analyzing data related to sociodemographic and sleep-related variables.

## 3. Results

The mean age of the child was found to be 15.28 ± 7.63 months. Around fifty-five percent of children were boys and 60.6% were the eldest in birth order. More than half (59.8%) of the respondents were educated to less than or up to high school. Majority of the respondents (84.3%) were mothers, and 79.5% of the respondents were homemakers. Seventy-seven percentage of respondents lived in the capital, Kathmandu, and 57.8% lived in a joint/extended family (Table 1).

Most (96%) of the children slept in the same bed with parents, and more than half (62.2%) of them slept in lateral position. The most common bedtime ritual (55%) was feeding (Table 2).

In the study, the mean duration of total sleep decreased with age. Similarly, there was a gradual decrease in duration of mean day time sleep and number and duration of night time awakening with age. The mean duration of night time sleep was almost the same throughout early childhood, i.e., 9.22 hours. There was variation in the mean duration of time required to put a child to sleep. Duration of total and day time sleep and number of night time awakening was found to be significantly associated with child's age(Table 3). The child on an average slept at around 21 pm in the night (not shown in the table).

According to BISQ criteria for sleep problems, 2% of children were found to be sleeping less than 9 hours, 16.86% woke up more than three times at night, and 0.80% woke up for more than one hour (Table 4).

Only 5.6% of respondents perceived that their children had sleep problems, and all of these respondents reported that the child only had small sleep problems (not shown in the table).

Table 5 shows that most parents perceived different sleep positions like prone sleeping, sleeping only on one side, as a sleep problem. Other sleep problems reported by the parents were screaming and sweating while sleeping, too much movement, short duration of sleep, late night sleeping, and taking long to fall asleep.

## 4. Discussion

The practice of cosleeping forms a natural part of parenting in eastern culture [16, 17], and the finding is similar in our study which showed that a large number of young children sleep with their parents in the same bed. The finding is further supported by the findings of a study done in India which reported that cosleeping is about 97.5% [6]. Though supine sleep position is generally recommended for infants to prevent the risk of sudden infant death syndrome (SIDS) [18], in our study, the most common sleeping position was found to be lateral. The lateral sleeping position has been reported to be the most common one in another study done in Nepal [11]. Most of the children in our study coslept with their parents, and research study suggest that there is no proven position for a child to sleep on in relation to their parents to prevent SIDS [19]. When cosleeping, factors like parental alcohol consumption, tiredness, smoking, use of duvets, and overcrowded living conditions are reported to be risk factors of SIDS rather than sleep position [19]. In Nepal, the infant mortality has largely been attributed to factors like birth intervals, type of assistance during child birth, and size of the baby [20]. There is almost no data on the prevalence of SIDS in Nepal.

Healthy bedtime rituals have been found to improve not only sleep but also enhance the overall growth and development of the child in early days [21]. More than half of the children in our study had feeding as a bed time ritual which is similar to a study finding where feeding has been considered a healthy and frequently used bed time ritual in young children [21].

Researches have shown that there are developmental changes in the sleep pattern as there occurs a decrease in daytime sleep and total sleep time with age and consolidation of sleep at night. Sleep consolidation is evidenced by a decrease in the frequency of night waking and nocturnal wakefulness [22]. A similar trend has been observed in our study as the mean total sleep duration and mean daytime sleep decreased as the child grew up. The decrease in mean total sleep duration with age is further supported by the study findings of another study [4]. The recommended sleep duration for an infant is 12-15 hours and that for toddlers is 11-14 hours [23]. Our study findings conform to the international recommendations as an infant included in our study, on an average, slept 12 hours and toddlers slept 11 hours.

Overall, 19.6% of the children had sleep problems in our study as per the BISQ parameter for sleep problems which is similar (20.3%) to the study findings of another study carried out in Lalitpur, Nepal [11]. However, only 5.6% of the respondents perceived that their children have sleep problems. This shows that there is a discrepancy between the prevalence of sleep problems among children and respondent's perception of it. A study carried out elsewhere has also reported that the parent reported sleep problem among young children may overlook relevant sleep symptoms and behaviors [10]. Studies have also shown that parents from Asian countries perceive more percentage of sleep problems in their children as compared to those from predominantly Caucasian countries [17].

Frequent night time awakening has been reported to lead to poor sleep quality, subjective feeling of insufficient sleep, and day time sleepiness in children [24]. In our study frequency of more than three night time awakening was found to be around 17% which is comparable to (13.9%) that reported in another study carried out in Nepal [11] while it is higher than the findings reported in another study carried out among school going children of China (9.8%) [24]. The difference in the findings of the studies may be because of the variation in sampling technique and study population. Children with total sleep duration of less than 9 hours were found to be 2% in the present study which is similar (2.7%) to another study carried out in Lalitpur, Nepal [11]. The duration of night time awakening of more than 1 hour was found to be 0.80% in our study which is very low as compared to 5.9% reported in another study [11]. The variation in these results could be due to random fluctuations.

The average time required to put a child to sleep was found to be 17 minutes in our study which is similar (19 minutes) to a finding from a systematic review carried out among children below two years of age [25]. The duration reported in our study is somehow comparable to another study carried out in Lalitpur, Nepal, where it was found to be 20 minutes on an average [11]. Studies have shown that Asian children sleep shorter duration [17, 25] and are put to sleep later than the Caucasian's; it was reported to be 19:27 pm in New Zealand while 22:17 pm in Hong Kong [17]. In our study, children, on an average, slept at 21 pm in the night which is similar (21:15 pm) to a study carried in Lalitpur, Nepal [11].

## 5. Conclusion and Recommendation

The study concludes that sleep problems exist among almost one-fifth of young children presenting to a tertiary healthcare facility in Kathmandu, but only few caregiver's are aware of it. Similarly, cosleeping and feeding as a bedtime ritual was common among children who participated in our study. Young children in our study were found to sleep less hours in total when compared to the children of same age groups in developed nations. Including sleep assessment as a part of every child's visit to the hospital is recommended to identify potential children with sleep problems.

### 5.1. Strengths and Limitations of the Study

The study looks into sleep characteristics and problems of children, which is a largely overlooked but important aspect of child health. However, the study was carried out in only one health care set up, so findings may not be representative of the actual problem nationwide. Recall bias may have occurred in data collection.

## Figures and Tables

**Table 1:** Sociodemographic characteristics of the respondents and child.

*n* = 249		
Sociodemographic variables	Frequency	Percentage
Mean age of the respondents = 28.67 ± 6.60 years		
Age of the children(in months)		
6-12	103	41.3
13-24	108	43.4
25-35	38	15.3
Mean age of the children = 15.28 ± 7.63 months		
Residence		
Kathmandu	192	77.1
Bhaktapur	36	14.5
Lalitpur	7	2.8
Outside of valley	14	5.6
Relationship with child		
Mother	210	84.3
Father	20	8.0
Grandparents	10	4.0
Others	9	3.6
Family type		
Nuclear	105	42.2
Joint/extended	144	57.8
Educational status of respondent		
Illiterate	8	3.2
Less than or equal to high school	149	59.8
Graduate	57	22.9
Postgraduate and higher	35	14.1
Occupation of respondent		
Home maker	198	79.5
Service	29	11.6
Business	17	6.8
Farming	2	0.8
Others	3	1.2
Sex of the child		
Male	136	54.6
Female	113	45.4
Birth order		
Eldest	151	60.6
Middle	3	1.2
Youngest	95	38.2

**Table 2:** Sleep characteristics of children.

*n* = 249		
Sleep characteristics	Frequency	Percentage
Place of sleep		
In the same bed with parents	239	96.0
Small cot/crib in same room with parents	8	3.2
In the same bed with grandparents	1	0.4
Others	1	0.4
Most common sleep position used		
Lateral	155	62.2
Supine	63	25.3
Prone	31	12.4
Bedtime rituals		
While feeding	137	55
Rocking	51	20.5
Lying in bed alone	25	10
Lying on bed along with parents	22	8.8
Carrying	14	5.6

**Table 3:** Sleep characteristics according to age group.

*n* = 249					
Sleep characteristics^∗^	Overall	6-12 months	13-24 months	25-35 months	^#^ *p* value
Duration of total sleep (hours)	12.12 ± 2.00	12.51 ± 2.30	11.99 ± 1.72	11.41 ± 1.64	0.013
Duration of night sleep (hours)	9.22 ± 1.19	9.22 ± 1.23	9.21 ± 1.10	9.23 ± 1.33	0.061
Duration of day sleep hours)	2.90 ± 1.66	3.29 ± 2.03	2.78 ± 1.27	2.17 ± 1.20	0.009
Duration of night time awakening (minutes)	6.65 ± 13.76	7.74 ± 14.88	6.25 ± 14.39	4.84 ± 7.30	0.209
Time to put to sleep (minutes)	17.27 ± 16.82	17.45 ± 16.22	16.95 ± 17.54	17.68 ± 16.78	0.987
Number of night time awakening (numbers)	2.10 ± 1.73	2.43 ± 1.95	2.01 ± 1.55	1.44 ± 1.34	0.007

^∗^Mean ± SD; ^#^*p* value significant at ≤ 0.05 for the Kruskal-Wallis *H* test.

**Table 4:** Children with sleep problems according to BISQ.

*n* = 249				
BISQ parameter^∗∗^	Overall (*n* = 249)	6-12 months (*n* = 103)	13-24 months (*n* = 108)	25-35 months (*n* = 38)
Sleep duration (less than 9 hrs)	5 (2.00)	2 (1.94)	1 (0.925)	2 (5.26)
Number of night waking(more than 3 times)	42 (16.86)	24 (23.30)	17 (15.74)	1 (2.63)
Duration of night waking(more than one hour)	2 (0.80)	1 (0.97)	1 (0.92)	—

^∗∗^Frequencies (percentages).

**Table 5:** Sleep problems reported by the parents.

*n* = 14		
Problems	Number	Percentage
Sleep position	6	42.85
Short duration of sleep	2	14.28
Frequent movement during sleep	1	7.14
Sweats during sleep	2	14.28
Screams in sleep	1	7.14
Sleeps late at night	1	7.14
Takes long to fall asleep	1	7.14

## Data Availability

Data will be made available on request.
